# Understanding Medical Decision-Making in Urogynecology: Insights from a Predominantly Hispanic Patient Population

**DOI:** 10.1007/s10903-026-01851-w

**Published:** 2026-01-16

**Authors:** Grace K. Sarris, Suyen Vilchez, Akshata Gunda, Christina Yarborough, Veronica Junco, Ariela Souroujon, Adam D. Williams, Katherine Amin, Alan J. Wein, Raveen Syan

**Affiliations:** 1 University of Miami Miller School of Medicine, Miami, USA; 2 Desai Sethi Urology Institute, University of Miami Hospital, Miami, USA

**Keywords:** Health literacy, Surgical decision making, Urogynecology, Social influences

## Abstract

To explore the role of health literacy in surgical decision making in a predominately Hispanic population, we designed a mixed-methods approach to assess patient’s literacy and explore what drives their choices for their healthcare. Understanding these factors will guide physicians to improve patient education, engagement and provide equitable care. In this IRB-approved study, English- and Spanish-speaking patients offered urogynecology surgery at our tertiary center were recruited. Participants completed the Short Assessment of Health Literacy (SAHL), a demographic survey, and a semi-structured interview to gain insight into their medical decision-making process. Data were analyzed using chi-square and t-tests, with *p* < 0.05 considered statistically significant. Grounded theory guided qualitative analysis of interview transcripts. Among 62 participants, most patients (90.3%) had sufficient health literacy. Mean SAHL scores did not differ between those electing for surgery and those declining. While Hispanic patients did tend to have lower SAHL scores (16.3) than non-Hispanic patients (17.1) (*p* = 0.04), the average still suggested adequate literacy. Interviews revealed that patients electing surgery often cited comfort with their physician (*p* = 0.01), definitive treatment (p = < 0.01), and friends’ experiences (*p* = 0.04) as influential factors. The influence of personal faith was brought up by nineteen participants (13 electing for surgery, 6 declining surgery, *p* = 0.11). Our research revealed that our patient population tends to have adequate health literacy. Interviews revealed themes that influence choices, such as trust in their physician and patient’s faith. Further investigation into the role that social factors play will be critical for better understanding patient surgical decision making.

**Supplementary Information:**

The online version contains supplementary material available at https://doi.org/10.1007/s10903-026-01851-w.

## Background

Pelvic floor disorders (PFDs) are increasingly prevalent globally with nearly one in four women over age twenty experiencing conditions such as urinary incontinence (UI), pelvic organ prolapse (POP), and overactive bladder (OAB) [1]. This prevalence increases to nearly 50% when looking at elderly populations over age eighty [2]. Concerningly, racial and ethnic disparities have been demonstrated in both treatment outcomes and research efforts: White women tend to be overrepresented in studies, despite data that supports Hispanic women often carry a disproportionate symptom burden [3]. Additionally, Hispanic women are also more likely to undergo surgery rather than a non-surgical intervention [4]. This difference in treatment among ethnicities emphasizes the need for further investigation into the factors that influence medical decision-making.

Health literacy profoundly impacts health outcomes, particularly in surgical care, where it affects pre- and post-operative adherence, pain management, recovery, and treatment satisfaction [5, 6]. Having adequate health literacy is multifaceted, involving a patient’s access to health information, along with their ability to interpret and apply this knowledge in medical-decision making [7]. Alarmingly, only one in eight adults in the United States (U.S.) demonstrate proficient health literacy, raising concerns about satisfactory patient understanding of their diagnoses and treatment options [5]. In urogynecology, research suggests that patients generally are health literate; however, these existing studies predominantly focus on English-speaking, educated White women, overlooking the diverse populations that face the highest PFD burden [8, 9]. Notwithstanding, Spanish-speaking women have been found to have limited comprehension of their diagnosis and treatment options, often relying on their physician to guide decision-making [10].

## Conceptual Framework

This study aims to elucidate influential factors in surgical decision-making, guided by the premise that these choices are shaped not only be health literacy. While being health literate provides the foundation for patients to process and understand information, it interacts within a broader context of psychological, social, and cultural influences. Our framework for this study integrates measurable health literacy and these social determinants to explain variation in decision-making for PFD treatment. The authors hypothesized that individuals with higher levels of health literacy would be more likely to elect for surgery but also anticipated that additional factors would significantly impact choices. By exploring decision-making within a wider context, our study aims to capture the complexity of surgical decision-making and provide insights specifically for underrepresented populations.

## Methods

### Participants

This study received approval from the University of Miami Institutional Review Board (IRB). Patients were recruited from an academic urology clinic between March and December 2024. Eligible participants were ≥ 18 years old, primarily English- or Spanish-speaking, and presented with urogynecology conditions for which both surgical and non-surgical treatment options were available. Exclusion criteria included illiteracy, significant cognitive impairment or psychiatric conditions affecting consent or participation, and prior surgery for the same diagnosis. Illiterate individuals were excluded as the objective measurement of health literacy required participants to be able to read. Patients were approached by their physician at their in-person appointments to ask if they would like to participate in a study aimed at evaluating health literacy and understanding the influences of medical decision making. Patients were told that involvement in the study would not affect their care. Participants received a $25 Amazon gift card as an honorarium following completion of all study-related activities. Recruitment of patients was designed to have similar numbers of English and Spanish-speaking patients to allow an analysis of potential differences in preferred language, as well as equal representation among those electing for and declining surgery. Total number of participants was limited by total funding available.

### Data Collection

Our primary outcome assessed is the various influential factors on patient’s decision making. Our secondary outcome was patient’s level health literacy, and the impact, if any, that had on their choices. Five female medical students (S.V., A.G., C.Y., V.J., and A.S.) with knowledge regarding PFDs were research assistants for this study. Students were instructed by a senior author (R.S.) who is an experienced researcher in qualitative studies. All coders were educated on how to conduct and subsequently analyze data elicited from qualitative interviews. The interviewer, introduced as a study team member, met the participant at the time of the interview and completed appropriate consents.

### Measures

Participants completed a demographic questionnaire capturing age, race, ethnicity, marital status, household size, income, education level, country of birth, menopause status, comorbidities, and diagnosis. Health literacy was objectively assessed using the Short Assessment of Health Literacy (SAHL), an 18-item tool validated in English and Spanish [11] (Supplemental Fig. 1). Participants were shown cards with medical terms and asked to identify related terms; scores ≥ 14 indicated adequate health literacy.

Semi-structured interviews were conducted by a research assistant fluent in the patient’s preferred language. Research assistants had a list of questions developed by the research team to guide interviews and explore patient understanding and decision making. All research assistants were appropriately trained to ensure competency and consistency among interviews. Interviews occurred in a private office in the clinic, with the option for a companion to be present. Discussions explored participants’ understanding of their diagnosis, treatment options, their understanding of treatment risks, and factors influencing their decision-making. Interviews were done once for each participant, with no repeat interviews taking place.

### Data Analysis

SAHL scores and demographic data were recorded in Microsoft Excel (Microsoft Corp., Redmond, WA), and statistical analyses were performed using SPSS v29 (IBM Corp., Armonk, NY). Categorical variables were analyzed using chi-square tests, with significance set at *p* < 0.05. Odds ratios (OR) and 95% confidence intervals (CI) were reported.

There were no notes taken during interview; however, they were audio-recorded. Recordings were securely uploaded to Box (Box Inc., Redwood City, CA), and transcribed using Microsoft Word (Microsoft Corp., Redmond, WA). Due to resource limitations, Spanish transcripts were translated into English by bilingual, native Spanish speakers on the research team. Transcripts were not returned to participants for review. Qualitative data were analyzed using thematic analysis to identify recurring themes elicited during interviews [12]. Three researchers (G.S., S.V., C.Y.) independently reviewed transcripts to identify recurring codes as they emerged and categorized them into overarching themes. Microsoft Word and NVivo (QSR International, Santa Clara, CA) were used for thematic organization. The team collaboratively refined thematic frameworks, integrating overlapping concepts for a comprehensive analysis to reduce subjectivity. Participants did not review the findings. Our methods and findings are reported within COnsolidated criteria for REporting Qualitative research (COREQ) 32 item Checklist.

## Results

A total of 62 urogynecology patients were included in the analysis. Among English-speaking patients, 17 elected for surgery, while 16 declined; of the Spanish-speaking patients, 16 elected for surgery, and 13 declined. Hispanic individuals comprised 67.8% of the study population, reflecting the minority-majority demographic served by our institution. Additionally, over two-thirds of participants were born outside the U.S. While most patients were aged 65 years or older, a notable proportion fell within the 35–64-year-old age range. Participant characteristics are reported in Table 1. Interviews lasted between 2 and 11 min (average duration 5.80 min).

**Table 1:** Patient characteristics by treatment choice

	All Patients (*n* = 62)	Elects for Surgery	Declines Surgery	*p*-value
**Age**				
18–24	2 (3.2%)	1 (50.0%)	1 (50.0%)	0.28
25–34	3 (4.8%)	2 (66.7%)	1 (33.3%)	
35–49	14 (22.6%)	10 (71.4%)	4 (28.6%)	
50–64	11 (17.7%)	8 (72.7%)	3 (27.8%)	
65+	32 (51.6%)	12 (37.5%)	20 (62.5%)	
**Race**				
Black	10 (16.1%)	6 (60.0%)	4 (40.0%)	0.39
White	40 (64.5%)	18 (45.0%)	22 (55.0%)	
Asian	1 (1.5%)	1 (100.0%)	0 (0.0%)	
Other	11 (16.9%)	8 (72.7%)	3 (27.3%)	
**Ethnicity**				
Hispanic	42 (67.8%)	21 (50.0%)	21 (50.0%)	0.40
Non-Hispanic	18 (29.0%)	11 (61.1%)	7 (38.9%)	
Prefer not to answer	2 (3.2%)	1 (50.0%)	1 (50.0%)	
**Birthplace**				
Born outside US	18 (29.0%)	9 (50.0%)	9 (50.0%)	0.30
Born in US	42 (67.8%)	22 (52.4%)	20 (47.6%)	
Other	2 (3.2%)	2 (100.0%)	0 (0.0%)	
**Education level**				
High school degree or less	13 (21.0%)	6 (46.2%)	7 (53.8%)	0.61
Some college	14 (22.6%)	6 (42.9%)	8 (57.1%)	
College degree	20 (32.3%)	12 (60%)	8 (40.0%)	
Advanced degree	12 (19.4%)	7 (58.3%)	5 (41.7%)	
Prefer not to answer	3 (4.8%)	2 (66.7%)	1 (33.3%)	
**Household income**				
<$20,000	10 (16.1%)	4 (40.0%)	6 (60.0%)	0.22
$20,000-$34,999	12 (19.4%)	5 (41.7%)	7 (58.3%)	
$35,000 to $49,999	7 (11.3%)	6 (85.7%)	1 (14.3%)	
$50,000 to $74,999	6 (9.7%)	4 (66.7%)	2 (33.3%)	
$75,000 to $99,999	5 (8.1%)	1 (20.0%)	4 (80.0%)	
>$100,000	5 (8.1%)	2 (40.0%)	3 (60.0%)	
Prefer not to answer	17 (27.4%)	11 (64.7%)	6 35.3%)	
**Relationship status**				
In a relationship	2 (3.2%)	0 (0.0%)	2 (100.0%)	0.39
Married	26 (41.9%)	14 (53.9%)	12 (46.1%)	
Single	20 (32.3%)	13 (65.0%)	7 (35.0%)	
Divorced	11 (17.7%)	5 (45.5%)	6 (54.5%)	
Prefer not to answer	3 (4.8%)	1 (33.3%)	2 (66.7%)	
**Menopause status**				
Pre-menopausal	18 (29.0%)	14 (77.8%)	4 (22.2%)	0.06
Post-menopausal	33 (53.2%)	16 (48.5%)	17 (51.5%)	
NA/prefer not to answer	11 (16.8%)	3 (27.3%)	8 (72.7%)	
**Number of people in household**				
1	11 (17.7%)	6 (54.5%)	5 (45.5%)	0.40
2–3	34 (54.9%)	16 (47.1%)	18 (52.9%)	
4–5	13 (21.0%)	7 (53.9%)	6 (46.1%)	
6+	3 (4.8%)	3 (100.0%)	0 (0.0%)	
Prefer not to answer	1 (1.6%)	1 (100.0%)	0 (0.0%)	
**Diagnosis**				
Overactive bladder	20 (32.3%)	13 (65.0%)	7 (35.0%)	0.14
Stress urinary incontinence	12 (19.4%)	6 (50.0%)	6 (50.0%)	
Prolapse	18 (29.0%)	11 (61.1%)	7 (38.9%)	
Other	12 (19.4%)	9 (75%)	3 (25%)	
**Type of patient**				
New patient	34 (54.9%)	20 (58.8%)	14 (41.2%)	0.62
Follow up	26 (41.9%)	12 (46.2%)	14 (53.8%)	
Unknown	2 (3.2%)	1 (50.0%)	1 (50.0%)	

### Quantitative Findings

Most participants (90.3%) demonstrated sufficient health literacy; only six scored in the low range (< 14), five of whom were Spanish-speaking and four were over age 60. Mean SAHL scores did not differ significantly between those electing surgery (16.41) and those who declined (16.68; *p* = 0.60). By race, White and Black patients had similar scores (16.77 vs. 17.22; *p* = 0.15), while Hispanic patients scored slightly lower than non-Hispanic participants (16.28 vs. 17.11; *p* = 0.04), though they were still considered to have sufficient literacy. English speakers had higher average scores than Spanish speakers (16.96 vs. 16.04; *p* = 0.11), but this was not significant. Further, education levels and household income were also not associated with differences in SAHL scores (*p* = 0.53 and *p* = 0.60, respectively).

### Qualitative Findings

Key themes relating to surgical decision-making emerged from patient interviews (Tables 2 and 3). Personal faith was a prominent factor, cited by 30.6% of participants (*n* = 19, 13 electing surgery, 6 declining, *p* = 0.11). The desire for definitive treatment was another dominant theme, mentioned by 29.0% of patients (*n* = 18) —16 of whom opted for surgery, while 2 declined (*p* < 0.01), suggesting a potential motivation. Many patients emphasized the importance of feeling comfortable with their physician, a sentiment shared by 24.2% of participants (*n* = 15), with a majority (13) electing surgery and only 2 who declined surgery (p = < 0.01).

**Table 2:** Influential factors for deciding treatment modality

	Number of patients who brought up theme			
Theme	Electing for surgery	Declining Surgery	Total	*P*-value
Seeking definitive treatment	16	2	18	**< 0.01**
Faith	13	6	19	0.11
Comfort with physician	13	3	15	**0.01**
Less invasive option	2	7	9	**0.04**
Friends with similar experience	7	1	8	**0.04**
Independent choice	7	2	9	0.11
Anesthesia	6	1	7	0.07
Hospital reputation	4	0	4	0.09
Concern with recovery time	3	1	4	0.37
Family planning	1	2	3	0.48
Prior negative surgical experience	0	3	3	0.06
Physical appearance	0	3	3	0.06
Familiar with medication	0	3	3	0.06

**Table 3:** Major themes elicited in patient interviews

Major Themes	Supporting Quotations
Patient’s comfort with their physician helps them to feel confident in their decisions.	“I like my doctor. I like the way she explained everything to me. She made me feel comfortable.”
	“Well, she made me feel comfortable as a patient. I thought she was very direct, well-spoken and listened.”
	“[I’m comfortable with] whatever she does because I know she’s the best. That’s why I made an appointment with her for like a year before, because I knew she’s the best of the best.”
	“What gives me security is that the doctor is the same doctor… I had surgery with her and well, the entire team, I felt very safe.”
Patient’s desire for definitive treatment drives their medical decision making.	“[to use] a pessary or to do anything else, it’s just like it’s just taking an Advil for a tumor in the brain… I want to fix the problem.”
	“I want security, no more wetting myself or wearing sanitary pads at night. Surgery feels like the most secure option. Other treatments require frequent applications, but I want something definitive.”
	“I have an option with the surgery. And [the vaginal prolapse] is going to be fixed, hopefully. That’s something I’m looking forward to. Forever fixed.”
	“[I choose surgery because] I want the most definitive solution. I’ve had this problem for three decades, and it’s gotten worse. I want something that will last.”
Patient’s faith influences their medical decisions.	“These are major decisions that I’m making, and I need guidance from God because I know eventually in the end, he knows what’s best for me.”
	“I’m Catholic and believe in God and the Virgin. Before coming here, I went to Mass and asked for guidance. I believe my prayers were answered.”
	“My faith… believing in God is first. Also, because I put Him on the alter, I put Him there, yes, it is His will that I have surgery.”
	“You just have to have faith sometimes…it’ll be okay, any kind of surgical procedure, you just pray on it.”

**Fig. 1:**
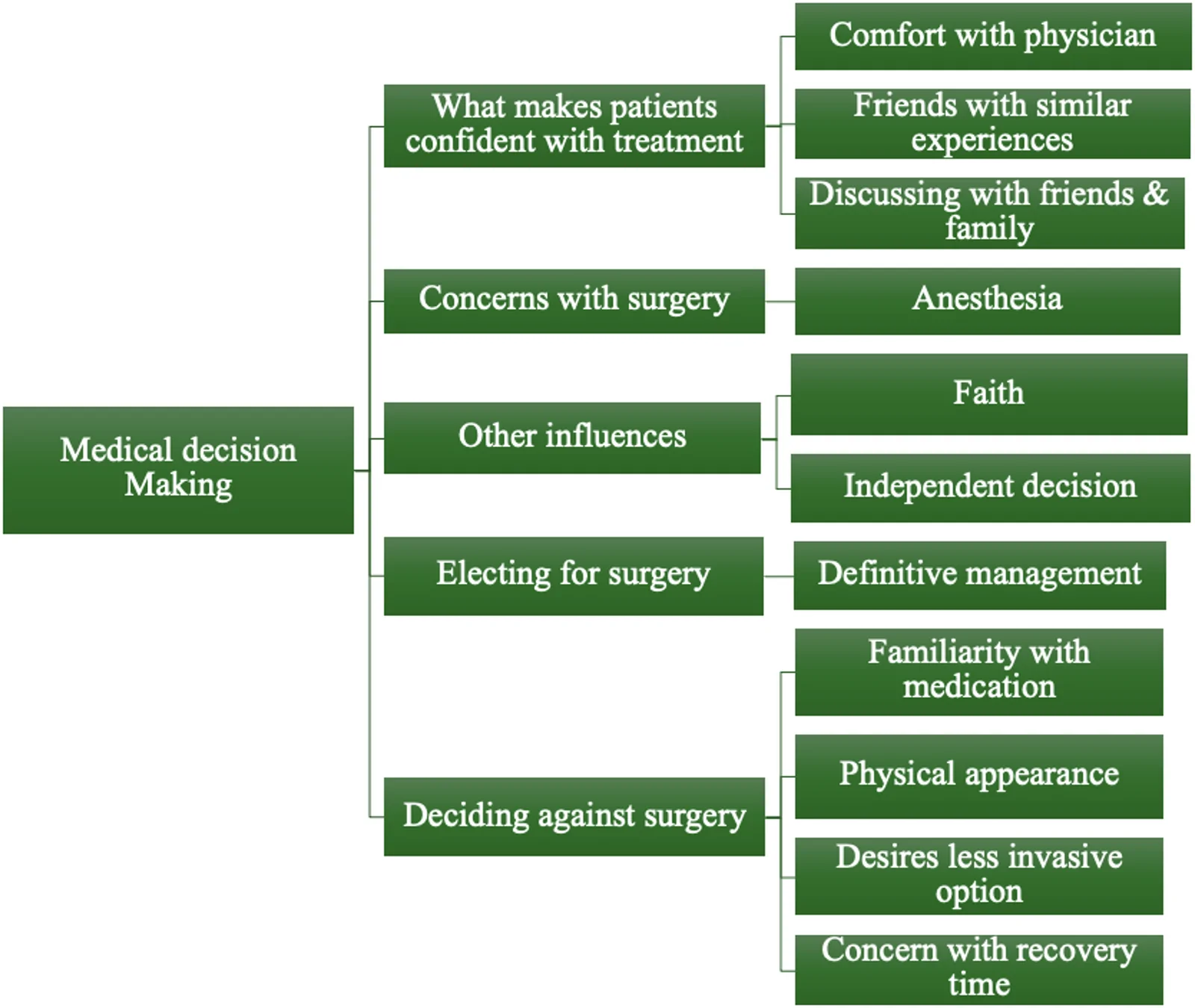
Coding tree of themes elicited during interviews

Having friends who had undergone treatment for PFDs was mentioned by 12.9% of patients. Of those eight patients, 87.5% (*n* = 7) were electing for surgery (*p* = 0.04), and only one who declined. Conversely, 14.5% of participants (*n* = 9, 7 electing for surgery, 2 declining, *p* = 0.11) reported making medical decisions independently of external opinions. Seven of these individuals had chosen surgical treatment, and two had declined (*p* = 0.11). Additional themes included concerns regarding anesthesia, acknowledged by seven participants, six of which were electing for surgery (*p* = 0.07). Concerns over recovery time was brought up by four patients, 75.0% (*n* = 3) of which were pursuing surgery (*p* = 0.37). The one theme mentioned only by patients choosing surgical treatment was the influence of the hospital’s reputation. Four patients brought this up (*p* = 0.09). Factors mentioned exclusively by patients declining surgery included prior negative surgical experiences (*n* = 3, *p* = 0.06), concerns over physical appearance (*n* = 3, *p* = 0.06), and familiarity with medication (*n* = 3, *p* = 0.06). The complete coding tree is outlined in Fig. 1.

## Discussion

Our study specifically examined a largely Hispanic population, where pelvic floor disorders are known to be highly common and bothersome [3]. Using a mixed-methods approach to capture diverse experiences, we explored what factors influence patient’s decisions when considering surgical management of PFDs. Understanding how both health literacy and social factors impact medical decision-making can guide more equitable, collaborative interventions.

Most participants demonstrated acceptable levels of health literacy, aligning with prior research [2, 9, 10]. We did not find a difference in health literacy scores among those choosing surgical versus non-surgical treatment. This contrasts the findings of Cayci et al., who observed an association between lower health literacy and surgery refusal [13]. This may be due to our tertiary care setting, where patients have often already seen other providers or attempted conservative treatments before seeking definitive surgical management. Similarly, age-related differences in health literacy were minimal. Only six participants in our study had low SAHL scores, suggesting inadequate health literacy. Of these six participants, four of them were over age 60—partially aligning with the trend that older adults are more vulnerable to low health literacy [2, 14]. This may reflect the urban setting of our institution, as rural populations have been shown to have lower health literacy [15, 16]. Hispanic participants had significantly lower average SAHL scores than non-Hispanic counterparts, however these scores still represented sufficient health literacy. While other studies suggest that socioeconomic factors among Hispanics, such as a poverty, health insurance, language barriers, this was not seen in our study [17]. Our institutional practices may offset this, as Spanish-speaking patients are generally seen by a fluent provider, or a certified medical interpreter.

The absence of an association between health literacy and socioeconomic status in our cohort suggests that income and education may play a less prominent role in our patient population than previously reported [18, 19]. This finding may be attributed to the tertiary care setting, where patients often have complex medical conditions, necessitating greater familiarity with the healthcare system. Additionally, increased access to medical information through the internet and social media may help bridge knowledge gaps, potentially mitigating the consequences of lower education levels. Platforms such as TikTok have been identified as an inexpensive and passive approach for improving health literacy [20]. More broadly, online resources have been shown to facilitate health-related information sharing, particularly among Latino/a individuals [21]. However, sociodemographic characteristics may play a role in who accesses these tools [22]. Future research should explore the true impact that these resources have on health literacy.

During interviews, the most prominent theme was patient’s faith, coming up in 19 interviews (30.6% of participants, 13 of which were electing for surgery and 6 declining). Patients interpreted faith broadly, encompassing religious, spiritual, or other personal beliefs. Notably, nearly one-third of participants acknowledged faith as a factor in their healthcare choices. Several individuals expressed that they would pray over their choices and have trust in a higher power to guide them to make the best decision. For instance, one individual reports “you got to trust in something…you know it’ll be okay, any kind of surgical procedure, you just pray on it”. Another participant shared a similar sentiment: “I pray to God. I tell him that it’s your decision to guide me into what’s best for me”. Another stated: “Before coming here, I went to Mass and asked for guidance. I believe my prayers were answered”. Reyes-Ortiz et al. reported that over 60% of Hispanic participants prayed for healing and considered spiritual well-being highly important, emphasizing religion’s relevance in this population [23]. Existing literature suggests that this may lead to discordance with medical recommendations, for instance, that increased spirituality may lead to lower interest in surgical treatment [23, 24]. Though our patient sample didn’t reveal the same patterns, faith still played a significant role. Being cognizant of these beliefs may help physicians to understand patient decision-making and guide culturally competent counseling.

Although some patients reported making their medical decisions independently (*n* = 9 (14.5%), 7 electing for surgery, 2 declining), many emphasized the role of social support (*n* = 8 (12.9%, 7 electing for surgery, 2 declining) and physician rapport (*n* = 15 (24.2%), 13 electing for surgery, 2 declining). Prior research highlights the significance of social networks, particularly among Hispanic patients [25]. Similarly, greater physician trust has been linked to increased confidence and comfort with to treatment plans [26]. Participants were asked what elements of their care made them feel comfortable with their treatment plan, and a common theme was their comfort with or confidence in their physician. A participant stated: “Number one [thing that makes me feel comfortable] is the doctor herself. How she explained everything to me”. Others mentioned specifically feeling listened to and having thorough explanations to the process: “Well [the doctor] made me feel comfortable as a patient. I thought she was very direct, well-spoken and listened” and “I like my doctor. I like the way she explained everything to me. She made me feel comfortable”. Our findings reinforce the value of interpersonal relationships in healthcare.

Taken together, these quotes highlight how feeling heard and well-informed directly influences patients’ confidence in their care. Still, these provider-patient dynamics represent are only part of the picture. Social networks can serve as valuable educational tools, and thus, normalizing these conversations is important for enhancing patient engagement and informed decision-making. Additional themes identified in patient interviews highlight the complexity of medical decision-making, including the interplay of personal preferences, perceived risks, and external influences. Logistical considerations, such as anesthesia-related fears, were commonly cited (*n* = 7 (11.2%), 6 electing for surgery, 1 declining), underscoring an opportunity for physicians to inquire about specific concerns, and to provide more targeted counseling to alleviate patient stress.

A key limitation of our study is the relatively small sample size, restricted by our funding structure. Another limitation of our study design is the structured format of the participant interviews. Interviewers did not consistently prompt participants to elaborate on their responses, limiting opportunities to explore their perspectives in greater depth. Additionally, these interviews were generally brief, and likely were not long enough to sufficiently explore this multi-factorial topic. The single-institution design further limits the generalizability of our findings, introducing potential sampling bias. This bias is compounded by the setting of a tertiary care center, which may attract individuals with higher health literacy than the general population. Due to the literacy requirements of the SAHL, we excluded illiterate patients, which may account for the low number of participants with limited health literacy. Future research could address this by employing alternative methods of assessing health literacy. Interviews were conducted by individuals meeting patients for the first time, which may have inhibited disclosure of personal motivations. Finally, transcript translations were performed by study team members rather than an impartial third party, introducing possible interpretive bias.

Despite these limitations, our study’s qualitative approach is a major strength, allowing participants to express nuanced perspectives and providing a deeper understanding of the complex factors influencing decision-making. The use of a validated tool to assess health literacy allowed us to feel confident in the categorization of patients being health literate or not. The diversity of our patient population allowed us to explore these themes across different groups.

## New Contribution To Literature

While our patient population tended to be health literate, our study adds to the literature by highlighting how medical knowledge intersects with cultural values and interpersonal dynamics in surgical decision-making for PFDs. In a predominantly Hispanic minority-majority community, we identified distinct influences shaping patient choices, including faith and their relationship with their physician. These findings expand on the current literature by emphasizing the importance of cultural context and social relationships. By uncovering these themes, our study provides a foundation for future research into how these factors shape surgical decision-making, as well as highlights topics for physicians to address when counseling patients, with the goal of providing culturally attuned models of patient-centered care.

## Supplementary Information

Below is the link to the electronic supplementary material.

Supplementary Material 1

## Data Availability

The data that support the findings of this study are available from the corresponding author upon request.
