# Supplementary material for: Understanding Medical Decision-Making in Urogynecology: Insights from a Predominantly Hispanic Patient Population
Source: J Immigr Minor Health. 2026 Jan 16;28(5):1088–96. doi: 10.1007/s10903-026-01851-w (PMC13612605; doi:10.1007/s10903-026-01851-w)
Supplement: Supplementary file 1 [file 10903_2026_1851_MOESM1_ESM.pdf]

The 18 items of *SAHL-E*, ordered according to item difficulty (keys and distracters are listed in the same random order as in the field interview)

**Stem**	**Key or Distracter**		
1. kidney	__urine	__fever	__don’t know
2. occupation	__work	__education	__don’t know
3. medication	__instrument	__treatment	__don’t know
4. nutrition	__healthy	__soda	__don’t know
5. miscarriage	__loss	__marriage	__don’t know
6. infection	__plant	__virus	__don’t know
7. alcoholism	__addiction	__recreation	__don’t know
8. pregnancy	__birth	__childhood	__don’t know
9. seizure	__dizzy	__calm	__don’t know
10. dose	__sleep	__amount	__don’t know
11. hormones	__growth	__harmony	__don’t know
12. abnormal	__different	__similar	__don’t know
13. directed	__instruction	__decision	__don’t know
14. nerves	__bored	__anxiety	__don’t know
15. constipation	__blocked	__loose	__don’t know
16. diagnosis	__evaluation	__recovery	__don’t know
17. hemorrhoids	__veins	__heart	__don’t know
18. syphilis	__contraception	__condom	__don’t know

Instruction for Administering *SAHL-E*

SHORT ASSESSMENT OF HEALTH LITERACY-ENGLISH (SAHL-E)
Interviewer's Instruction

The *Short Assessment of Health Literacy-English*, or *SAHL-E,* contains 18 test items designed to assess an English-speaking adult's ability to read and understand common medical terms. The test could help health professionals estimate the adult's health literacy level. Administration of the test could facilitated by using laminated 4"×5" flash cards, with each card containing a medical term printed in boldface on the top and the two association words—i.e., the key and the distracter—at the bottom.

Directions to the Interviewer:

1 Before the test, the interviewer should say to the examinee:
"I'm going to show you cards with 3 words on them. First, I'd like you to read the top word out loud. Next, I'll read the two words underneath and I'd like you to tell me which of the two words is more similar to or has a closer association with the top word. If you don't know, please say 'I don't know'. Don't guess."

2. Show the examinee the first card.

3. The interviewer should say to the examinee:
"Now, please, read the top word out loud."

4. The interviewer should have a clipboard with a score sheet to record the examinee's answers. The clipboard should be held such that the examinee cannot see or be distracted by the scoring procedure.

5. The interviewer will then read the key and distracter (the two words at the bottom of the card) and then say:
"Which of the two words is most similar to the top word? If you don't know the answer, please say 'I don't know'."

6. The interviewer may repeat the instructions so that the examinee feels comfortable with the procedure.

7. Continue the test with the rest of the cards.

8. A correct answer for each test item is determined by both correct pronunciation and accurate association. Each correct answer gets one point. Once the test is completed, the interviewer should tally the total points to generate the *SAHL-E* score.

9. A score between 0 and 14 suggests the examinee has low health literacy.
